# Prevalence and factors associated with characteristics of hepatitis B susceptibility among vaccinated adults in Malaysia: a cross-sectional study

**DOI:** 10.1038/s41598-026-42115-9

**Published:** 2026-03-03

**Authors:** Filza Noor Asari, Eida Nurhadzira Muhammad, Nurfatehar Ramly, Mohd Hatta Abdul Mutalip, Muhammad Faiz Mohd Hisham, Zhuo Lin Chong

**Affiliations:** Institute for Public Health, National Institutes of Health, Setia Alam, Selangor Malaysia

**Keywords:** Hepatitis B, Susceptibility, Prevalence, Vaccinated adults, Malaysia, Diseases, Gastroenterology, Health care, Medical research, Risk factors

## Abstract

Hepatitis B vaccination is a cornerstone of global disease prevention, yet susceptibility among vaccinated individuals remains a concern. This study aimed to determine the prevalence and factors associated with hepatitis B susceptibility in vaccinated adults in Malaysia. A cross-sectional analysis was conducted using data from the National Health and Morbidity Survey (NHMS) 2020, involving 4,083 respondents aged 15 years and older. Serological testing for hepatitis B virus (HBV) markers, HBsAg, anti-HBs, and anti-HBc assays was performed. Susceptibility was defined based on serological profiles, and complex sampling logistic regression was employed to identify associated risk factors. Among the participants, 64.9% (2,649/4,083) were susceptible to HBV infection, with a prevalence of 22.9% among vaccinated individuals. Susceptibility was notably higher in young adults aged 15–29 (19.0%), and Malay ethnic group (20.5%). Additionally, individuals with tertiary education (18.9%) and single marital status (20.1%) exhibited elevated susceptibility. Multivariate analysis revealed significant associations with ethnicity, where Chinese (aOR 7.10, 95% CI: 2.67, 18.91) and other ethnic groups (aOR 5.67, 95% CI: 2.03, 15.92) had higher odds of susceptibility compared to Malays. Education level was another strong predictor, with tertiary (aOR 9.82, 95% CI: 3.61, 26.73), secondary (aOR 8.88, 95% CI: 3.42, 23.11), and primary education (aOR 6.96, 95% CI: 2.77, 17.51) showing substantially increased odds relative to no formal education. This study highlights a substantial prevalence of hepatitis B susceptibility among vaccinated adults in Malaysia. Ethnicity and education level emerged as significant factors. These findings underscore the need for targeted interventions, such as booster vaccinations or enhanced screening, particularly for high-risk groups like the Chinese and other minority ethnicities, as well as those with higher education levels. Further research is warranted to explore these associations’ underlying reasons and optimize vaccination strategies in Malaysia.

**Supplementary Information:**

The online version contains supplementary material available at 10.1038/s41598-026-42115-9.

## Introduction

Hepatitis B virus (HBV) infection remains a major global public health concern, with an estimated 254 million people living with chronic HBV in 2022 and 1.2 million new infections annually. Chronic infection is a leading cause of liver cirrhosis and hepatocellular carcinoma (HCC), contributing to nearly 1.1 million deaths annually worldwide^1,2^. The availability of a safe and effective vaccine since 1982 has markedly reduced HBV transmission, particularly in countries that have adopted universal infant vaccination.

Despite decades of progress in HBV prevention, global elimination remains a distant goal. With only six years left to meet the World Health Organization (WHO) 2030 targets, many countries, particularly low and middle-income nations that carry the highest burden of chronic hepatitis B (CHB), continue to face substantial challenges^3^.

Malaysian was among the first countries to implement a universal newborn hepatitis B vaccination program in 1989, which has successfully reduced HBV prevalence in children and adolescents^4,5^. Despite these achievements, recent national and regional studies indicate that a significant proportion of vaccinated adults remain susceptible to HBV infection^6^. Sero surveillance studies have reported varying levels of susceptibility across different age groups, ethnicities, and education levels, indicating that demographic and social determinants play a critical role in influencing long-term immunity^7^. Understanding this phenomenon is essential to inform both policy and clinical practice.

Several mechanisms may explain susceptibility despite prior vaccination. These include waning immunity over time, whereby protective anti-HBs antibody levels fall below the threshold of 10mIU/mL in adulthood, leaving individuals vulnerable to infection^8,9^. Additionally, a proportion of individuals are primary vaccine non-responders, failing to mount adequate immune protection even after completing the full vaccination schedule^10^. Other contributing factors include incomplete vaccination, programmatic gaps in earlier years of rollout, and the possible role of vaccine escape mutants, which can evade vaccine-induced immunity^11^.

The World Health Organization’s 2022 guidelines emphasise the importance of ongoing sero surveillance and booster considerations in specific high-risk groups, despite not recommending universal adult boosters^1^. Similarly, the Malaysian Ministry of Health National Immunisation Programme (NIP) underscores the success of infant HBV immunisation but acknowledges that immunity gaps in older adolescents and adults, especially those vaccinated in infancy may persist, necessitating targeted interventions^5,12^.

Given these concerns, there is an urgent need to examine susceptibility patterns among vaccinated adults in Malaysia. Identifying the sociodemographic and clinical factors associated with loss of immunity will help policymakers and clinicians refine hepatitis B control strategies, strengthen surveillance, and align national programs with the WHO’s 2030 goal of eliminating viral hepatitis as a public health threat^13^.

## Methods

### Sampling frame and design

We analysed data from NHMS 2020, a nationally representative, two-stage stratified cluster random sample of Malaysians residing in non-institutional living quarters. Primary stratification was by state/Federal Territory; secondary stratification distinguished urban vs. rural areas within each state. In stage 1, enumeration blocks were selected with probability proportional to size; in stage 2, living quarters were randomly selected within each block. All analyses applied design weights, non-response adjustments, and post-stratification to national population controls, and used complex-survey procedures to obtain valid estimates and standard errors.

### Sample size and statistical power

NHMS 2020 master sample was determined to achieve nationally representative precision targets under a complex design. For the HBV serology module, 4,083 participants provided analysable specimens (Supplementary Table S1). We report design-based 95% confidence intervals for all prevalence estimates to reflect actual precision. At the national level, precision was adequate (e.g., overall HBV susceptibility 62.9% (95% CI 57.4–68.0); we acknowledge wider CIs for subgroup estimates among vaccinated adults due to smaller effective sample sizes, and we interpret these with appropriate caution.

### Inclusion and exclusion criteria

Inclusion criteria were: age ≥ 15 years, residence in selected living quarters, provision of written informed consent/assent, successful phlebotomy with sufficient serum, and complete HBV marker results (HBsAg, anti-HBs, anti-HBc). We excluded individuals without valid serology or with indeterminate/missing HBV marker results.

### Definition of susceptibility and vaccinated sub-cohort

HBV susceptibility was defined per CDC-aligned serologic criteria: HBsAg negative, anti-HBc negative, and anti-HBs < 10 *mIU/mL*^14^. The vaccinated adult sub-cohort comprised respondents reporting any prior HBV vaccination, verified by vaccination card/medical record where available. This allowed us to evaluate susceptibility among those with a vaccination history, while the primary susceptibility definition remained serology-based.

### Verification of vaccination status

Vaccination history was obtained via face-to-face interview using a structured questionnaire adapted from NHANES^15^, with verification from vaccination cards or medical records when available. We applied a hierarchy of evidence (documented record > registry notation if available > self-report).

### Biospecimen collection and serological analysis

Data collection was conducted between August 7 and October 11, 2020. Informed consent was obtained from participants aged 18 years and above, while assent and parental or guardian consent were secured for those aged 15–17 years, in accordance with Malaysian ethical guidelines for research involving minors.

Participants were interviewed to obtain demographic, clinical, and epidemiological information, followed by venous blood collection. A 5.0 mL sample was drawn into a gel-separation tube by trained medical personnel under aseptic conditions. To ensure biospecimen integrity, samples were immediately centrifuged on-site using portable centrifuge units to separate serum, minimizing hemolysis and analyte degradation. The serum was then stored in portable chillers maintained at 2–8 °C with continuous temperature monitoring^16^.

Serological testing for hepatitis B virus (HBV) markers was performed in a certified clinical laboratory using standardized, high-sensitivity immunoassays. Hepatitis B surface antigen (HBsAg) was detected using the Architect HBsAg Qualitative 11 Assay (Abbott Laboratories) based on the Chemiluminescent Microparticle Immunoassay (CMIA) platform. Hepatitis B surface antibody (anti-HBs) was quantified using Alinity 1 anti-HBs Assay, while total hepatitis B core antibody (anti-HBc Total) and IgM hepatitis B core antibody (IgM anti-HBc) were determined using the Architect anti-HBc Total and Architect anti-HBc IgM Assays, respectively. All assays were performed according to manufacturer protocols and internal quality control procedures to ensure analytical accuracy and consistency.

### Statistical analysis

Descriptive and inferential analyses were conducted using complex sample procedures to account for the survey’s two-stage stratified cluster sampling design. Sampling weights were applied to generate nationally representative estimates, incorporating adjustments for unequal selection probabilities, non-response, and post-stratification to align with national population projections. Prevalence estimates with design-based 95% confidence intervals (CIs) were calculated to reflect the true precision of population parameters. Logistic regression analysis, using complex sampling methods, was employed to identify factors independently associated with hepatitis B susceptibility among vaccinated individuals (Supplementary Table S2). Variables entered into the model were selected based on biological plausibility, previous evidence, and data completeness, including age group, ethnicity, education level, and marital status. Model diagnostics were conducted to assess fit and reliability. Adjusted odds ratios (aORs) and corresponding 95% CIs were reported, with statistical significance defined as *p* < 0.05 (Supplementary Table S2). All analyses were performed using IBM SPSS Statistics version 26.

### Ethical considerations

The survey was registered with the National Medical Research Registry as NMRR-190867-47973. The protocol and the procedures used were approved by the Medical Research and Ethics Committee (MREC), Ministry of Health, which operates in accordance with the International Council for Harmonization of Technical Requirements for Pharmaceuticals for Human Use (ICH), the Malaysian Guidelines for Good Clinical Practice, and the ethical principles outlined in the Declaration of Helsinki. All participants provided written informed consent before being recruited into the study.

## Results

### Overall prevalence of HBV susceptibility

Of the 4,083 adults tested, 62.9% (95% CI 57.4–68.0) were susceptible to HBV infection, representing an estimated population of 15,155,978 adults aged ≥ 15 years (Supplementary Table S1).

### Age-specific trends

**Fig. 1:**
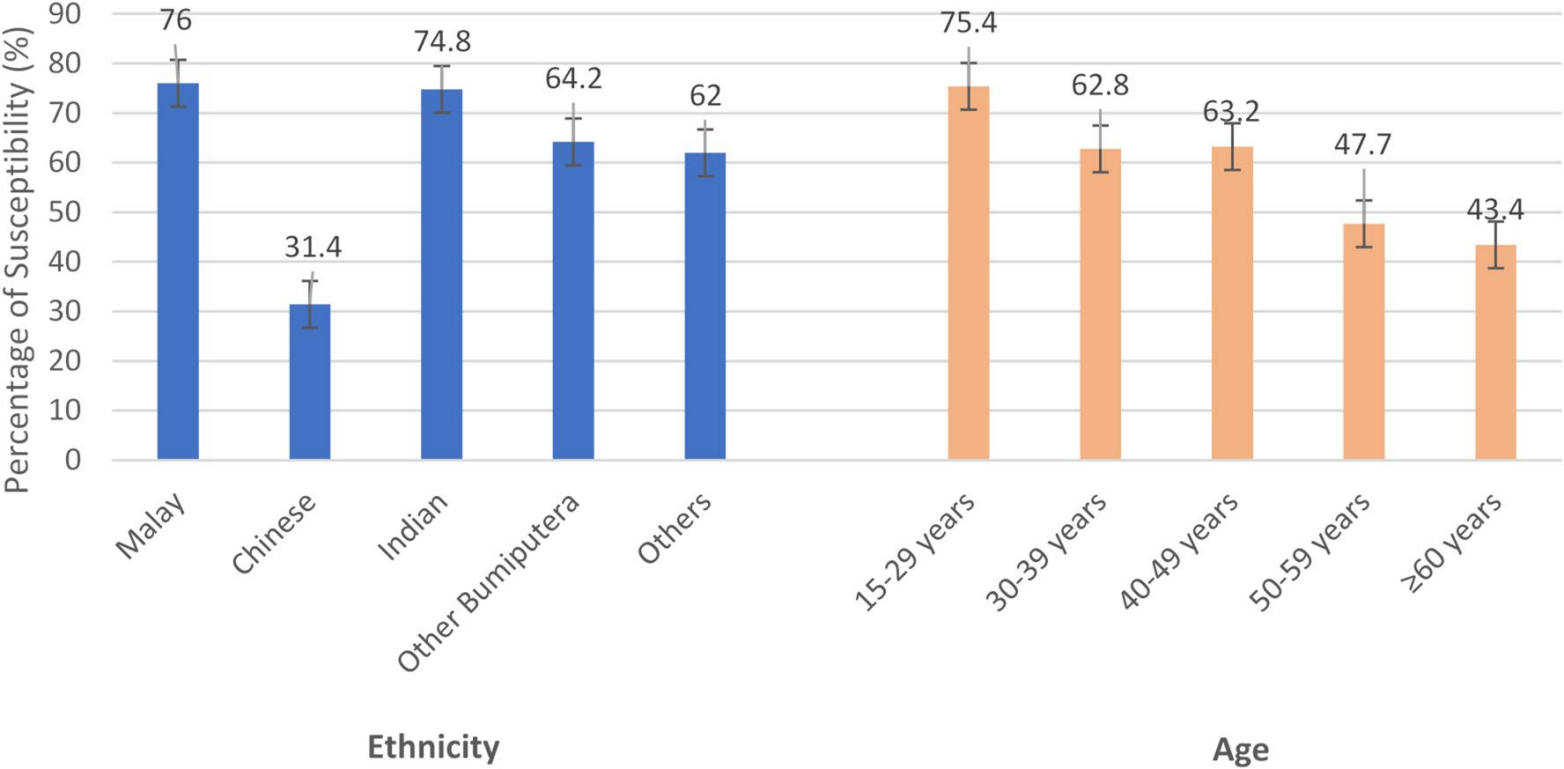
Prevalence of susceptibility to HBV by age-specific group and ethnicity among Malaysian adults.

A strong age gradient was evident (*p* < 0.001). Susceptibility was highest in young adults aged 15–29 years 75.4% (95% CI 67.6–81.8), and declined steadily with age 62.8% (95% CI 55.6–69.5) in 30–39 years, 63.2% (95% CI 55.3–70.4) in 40–49 years, 47.7% (95% CI 40.6–55.0) in 50–59 years, and lowest at 43.4% (95% CI 37.0-50.1) among those ≥ 60 years (Fig. 1). This pattern indicates that younger cohorts-despite being the first benefit from Malaysia’s universal newborn vaccination program introduced in 1989-remain the most vulnerable, likely reflecting waning vaccine-induced immunity or incomplete vaccination. By contrast, the lower susceptibility in older adults (≥ 60 years) likely reflects natural immunity from prior exposure, consistent with their higher prevalence of anti-HBc positivity.

### Sociodemographic patterns

Marked ethnic disparities were observed. Malays showed the highest susceptibility 76% (95% CI 72.2–79.5), whereas Chinese had the lowest prevalence of susceptibility 31.4% (95% CI 23.6–40.4). Indians 74.8% (95% CI 64.5–82.9) and Other Bumiputera 64.2% (95% CI 56.4–71.4) also demonstrated high susceptibility (Fig. 1).

By education level, susceptibility was most common among those with secondary education 67.2% (95% CI 61.4–72.6), and least common in those with primary education only 52.5% (95% CI 45.1–59.8). Interestingly, adults with tertiary education had persistently elevated susceptibility 64% (95% CI 56.6–70.9) (Table 1), a counterintuitive finding given their presumed greater health literacy.

Marital status also influenced susceptibility: single adults 71.8% (95% CI 65.3–77.6) were substantially more susceptible than married 60.7% (95% CI 55.4–65.7) or widow(er)/divorcee individuals 44.1% (95% CI 34.8–53.8) (Table 1). These patterns may reflect differences in health-seeking behavior, vaccine uptake, or exposure risk.

### Susceptibility among vaccinated adults

**Fig. 2:**
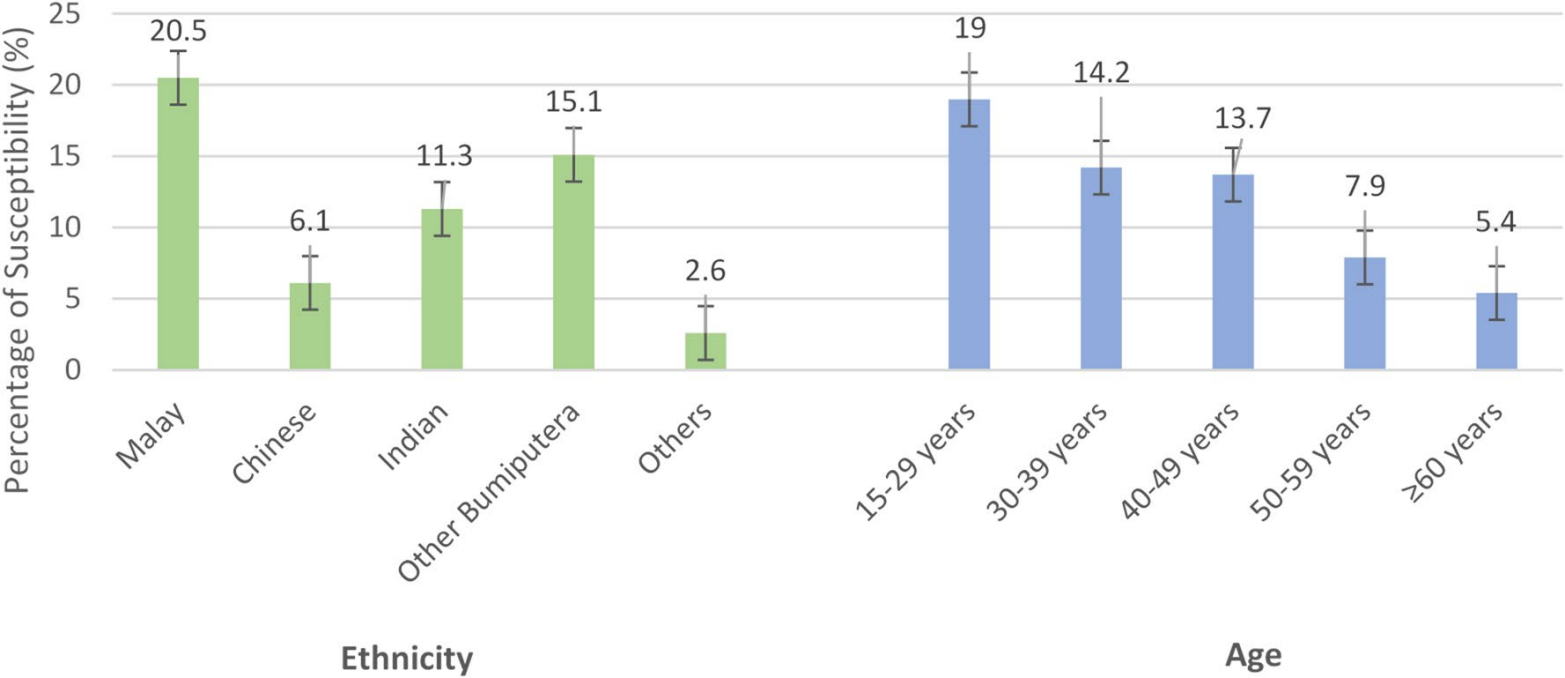
Prevalence of susceptibility to HBV by age-specific group and ethnicity among Malaysian vaccinated adults.

Among those who reported prior HBV vaccination, 22.9% (95% CI 16.8–30.5) remained susceptible. Young adults aged 15–29 years were most affected 19.0% (95% CI 13.5–26.2), followed by those aged 30–39 years 14.2% (95% CI 10.2–19.4). In contrast, susceptibility was uncommon among vaccinated adults ≥ 60 years 5.4% (95% CI 3.3–8.6), reinforcing the possibility that older individuals benefited from naturally acquired immunity in addition to vaccination. By ethnicity, susceptibility was highest among Malays 20.5% (95% CI 14.1–28.8), followed by other Bumiputera 15% (95% CI 10.3–21.5). Chinese had the lowest susceptibility 6.1% (95% CI 3.3–11.1), despite being at higher adjusted odds of susceptibility in regression models (Fig. 2).

**Table 1:** Prevalence of susceptibility to HBV by sociodemographic variables among Malaysian adults and among vaccinated adults.

Variables	Susceptible to Hepatitis B		Susceptible to Hepatitis B among vaccinated adults	
	Count, *n*	Prevalence (%)	Count, *n*	Prevalence (%)
Overall	2649	62.9 (57.4, 68.0)	548	22.9 (16.8, 30.5)
Residential area				
Urban	1478	63.4 (56.7, 69.5)	346	15.0 (10.3, 21.5)
Rural	1171	61.1 (52.9,68.6)	202	9.8 (5.4, 16.9)
Gender				
Male	1198	61.6 (56.0, 67.0)	249	13.4 (9.4, 18.8)
Female	1451	64.2 (58.2,69.8)	299	14.3 (10.2, 19.8)
Age group				
15–29	979	75.4 (67.6, 81.8)	265	19.0 (13.5, 26.2)
30–39	533	62.8 (55.6, 69.5)	122	14.2 (10.2, 19.4)
40–49	442	63.2 (55.3, 70.4)	80	13.7 (7.9, 22.7)
50–59	348	47.7 (40.6, 55.0)	48	7.9 (4.1, 14.7)
60 years and above	347	43.4 (37.0, 50.1)	33	5.4 (3.3, 8.6)
Ethnicity				
Malay	1807	76.0 (72.2, 79.5)	379	20.5 (14.1, 28.8)
Chinese	111	31.4 (23.6, 40.0)	24	6.1 ((3.3, 11.1)
Indian	155	74.8 (64.5, 82.9)	19	11.3 (5.1, 22.9)
Other Bumiputera^a^	429	64.2 (56.4, 71.4)	117	15.1 (9.2, 23.9)
Others	147	62.0 (47.4, 74.7)	9	2.6 (1.0, 6.5)
Education level				
No formal education	123	57.6 (40.4, 73.1)	7	5.8 (0.8, 32.7)
Primary education	488	52.5 (45.1, 59.8)	71	7.5 (5.0, 11.1)
Secondary education	1344	67.2 (61.4, 72.6)	294	15.2 (10.9, 20.8)
Tertiary education	694	64.0 (56.6, 70.9)	176	18.9 (13.0, 26.7)
Marital status				
Single	877	71.8 (65.3, 77.6)	245	20.1 (14.2, 27.5)
Married	1584	60.7 (55.4, 65.7)	281	11.6 (8.1, 16.4)
Widow(er)/Divorcee	187	44.1 (34.8, 53.8)	22	6.1 (3.4, 10.9)

Note:

^a^ Other Bumiputera includes Bumiputera Sabah, Bumiputera Sarawak and Orang Asli.

Tertiary-educated adults again stood out, with 18.9% (95% CI 13.0-26.7) susceptibility despite vaccination. Single adults were also more affected 20.1% (95% CI 14.2–27.5) compared to their married or widowed/divorced counterparts (Table 1).

### Multivariate associations

After adjusting for sociodemographic factors, several variables remained significantly associated with Hepatitis B susceptibility (Table 2). Age demonstrated a strong protective effect, with adults aged ≥ 60 years having significantly lower odds of susceptibility compared with those aged 15–29 years (aOR 0.38, 95% CI 0.22–0.69, *p* = 0.001). In terms of ethnicity, Chinese adults exhibited markedly higher odds of susceptibility, with a more than sevenfold increase compared to Malays (aOR 7.10, 95% CI 2.67–18.91, *p* < 0.001). Other minority ethnic groups also showed significantly elevated odds of susceptibility (aOR 5.68, 95% CI 2.03–15.92, *p* = 0.001).

Educational attainment was strongly associated with hepatitis B susceptibility, demonstrating a clear dose-response relationship. Individual with tertiary education had nearly tenfold higher odds of susceptibility compared with those with no formal education (aOR 9.82, 95% CI 3.61–26.73), while those with secondary and primary education also showed increased odds of susceptibility relative to reference group (Table 2). Marital status was another significant factor, whereby individuals who were widowed or divorced had lower odds of susceptibility compared with those who were single (aOR 0.54, 95% CI 0.31–0.94, *p* = 0.029).

**Table 2:** Sociodemographic factors associated with HBV susceptibility among vaccinated adults (Univariate & Multivariate logistic regression).

	Susceptible & vaccinated			
	Univariate analysis		Multivariate analysis	
	OR (95% CI)	p value	aOR (95% CI)	p value
Residential				
Urban	*1.00 (Ref)*		*1.00 (Ref)*	
Rural	0.61 (0.28, 1.32)	0.206	0.52 (0.24, 1.10)	0.087
Age group (years)				
15–29	*1.00 (Ref)*		*1.00 (Ref)*	
30–39	0.70 (0.49, 0.99)	0.044	0.79 (0.54, 1.17)	0.240
40–49	0.67 (0.41, 1.10)	0.115	0.81 (0.49, 1.32)	0.391
50–59	0.36 (0.21, 0.62)	< 0.001	0.47 (0.25, 0.90)	0.240
60 and above	0.24 (0.15, 0.38)	< 0.001	0.38 (0.22, 0.69)	0.001
Ethnicity				
Malay	*1.00 (Ref)*		*1.00 (Ref)*	
Chinese	9.73 (3.39, 27.9)	< 0.001	7.10 (2.67, 18.91)	< 0.001
Indian	2.45 (0.62, 9.72)	0.201	1.70 (0.43, 6.72)	0.445
Other Bumiputra^a^	4.78 (1.23, 18.51)	0.024	3.40 (0.86, 13.42)	0.080
Others	6.70 (2.28, 19.68)	< 0.001	5.68 (2.03, 15.92)	0.001
Education				
No formal education	*1.00 (Ref)*		*1.00 (Ref)*	
Primary education	10.17 (4.00, 25.81)	< 0.001	6.96 (2.77, 17.51)	< 0.001
Secondary education	22.53 (8.19, 61.94)	< 0.001	8.88 (3.42, 23.11)	< 0.001
Tertiary education	29.32 (9.77, 88.10)	< 0.001	9.82 (3.61, 26.73)	< 0.001
Marital status				
Single	*1.00 (Ref)*		*1.00 (Ref)*	
Married	0.53 (0.40, 0.69)	< 0.001	0.73 (0.52, 1.01)	0.060
Widow(er)/Divorcee	0.26 (0.16, 0.43)	< 0.001	0.54 (0.31, 0.94)	0.029

Note:

^a^ Other Bumiputera includes Bumiputera Sabah, Bumiputera Sarawak and Orang Asli.

## Discussion

This nationwide study provides important evidence on hepatitis B virus (HBV) susceptibility among Malaysian adults, highlighting that a substantial proportion remain vulnerable to infection despite the country’s long-standing vaccination program. While Malaysia introduced universal infant vaccination in 1989^5^, our findings show that protective immunity in adulthood is not assured, particularly among younger cohorts vaccinated in infancy, certain ethnic groups, and those with higher education levels.

When compared with international data, the overall susceptibility rate of 62.9% in Malaysia is somewhat lower than the 73.4% reported in the United States, but higher than in several East Asian settings where booster strategies or intensive catch-up programs have been implemented^17^. Notably, in China and South Korea, studies have demonstrated more durable immunity due to broader adult vaccination programs and periodic serological monitoring^18^. By contrast, in Southeast Asia, uneven implementation of adult vaccination and reliance on childhood immunization have led to heterogeneous susceptibility profiles^19^, which Malaysia appears to reflect.

Our results suggest that waning immunity is a likely contributor, particularly among those aged 15–29 years who were born after the introduction of the infant vaccination program. Similar findings have been documented in Taiwan and Thailand, where anti-HBs levels declined sharply after adolescence^6,20^. Research revealed that although many had been vaccinated in childhood, a substantial proportion lacked protective antibody levels in adulthood^20^. Another study reported that 72.4% of medical students born between 1991 and 1995 had anti-HBs levels below 10mIU/ml^6^.

Beyond waning immunity, biological mechanisms may also contribute to persistent susceptibility. Host genetic factors, including variations in human leukocyte antigen (HLA) alleles such as HLA-DRB1 and HLA-DQB1, as well as cytokine polymorphisms, have been associated with reduced antibody response and diminished durability of protection following hepatitis B vaccination^10,21^. Similarly, cytokine gene polymorphisms, including those affecting IL-10 and TNF-α, have been implicated in weaker immune responses^22^. These genetic variations may partially explain why some ethnic groups, such as Chinese and other minorities in our study, demonstrated higher odds of susceptibility despite reported vaccination.

Behavioral and structural mechanisms are equally important. Individuals with higher education levels, although expected to have better health literacy, may paradoxically exhibit lower vaccine follow-up due to misplaced confidence in their health status, competing priorities, or skepticism toward the need for boosters^23^. Misconceptions that a completed childhood vaccination series provides lifelong immunity, or fears about vaccine side effects, may also contribute to gaps in adult protection^24^. Furthermore, single adults may have lower healthcare utilization due to reduced family or social support structures, which are strong determinants of preventive health behaviors^25^. They may also engage more frequently in risk behaviors such as multiple sexual partnerships, inconsistent condom use, or substance use, which heighten exposure risk to HBV^24^.

These findings carry several policy implications. First, routine serological screening of adults, especially those in high-risk occupational or demographic groups, could identify individuals with waning immunity. Second, booster vaccination policies should be considered for young adults and vulnerable subgroups. Third, culturally tailored outreach strategies are needed to reduce ethnic disparities, particularly by engaging community leaders and improving health literacy. Finally, greater attention to unmarried adults as a potentially neglected group in preventive health could reduce vulnerability.

### Strengths and limitations

This study has several notable strengths. It is based on data from a large, nationally representative sample derived from the NHMS 2020, employing a robust two-stage stratified cluster sampling design. This enhances the generalizability of the findings to the Malaysian adult population. The study also utilized laboratory-confirmed serological testing (HBsAg, anti-HBs, and anti-HBc) to objectively define hepatitis B susceptibility, reducing the risk of misclassification associated with self-reported vaccination status. Additionally, complex sampling weights were applied in all analyses to ensure national representative estimates, and multivariate regression allowed for the identification of independent risk factors while adjusting for the confounders.

However, some limitations must be acknowledged. First, the response rate for HBV testing was 77.0%, slightly below the commonly accepted 80% threshold. This introduces the potential for non-response bias, as individuals who declined testing may differ systematically from those who participated. Secondly, there is a possibility of misclassification bias, as some individuals who were previously vaccinated may have undetectable levels of anti-HBs due to waning immunity over time. This could have led to an overestimation of hepatitis B susceptibility in the study population. Thirdly, while serological testing provides objective information on immune status, data on vaccination history and exposure risks were based on self-report and may be subject to recall bias or missing data.

Future research should focus on longitudinal cohort studies to track immunity persistence, intervention trials to evaluate the effectiveness of booster strategies, and surveillance systems to monitor HBV susceptibility trends across demographic subgroups. Such evidence will be crucial as Malaysia works toward the WHO’s 2030 goal of eliminating viral hepatitis as a public health threat.

## Conclusions

This nationwide study demonstrates that a substantial proportion of Malaysian adults remain susceptible to hepatitis B, including a notable share of previously vaccinated individuals, indicating waning immunity over time. Younger age, higher education levels, and ethnic disparities emerged as key risk factors, underscoring that universal vaccination in infancy alone is insufficient to ensure long-term protection.

To strengthen hepatitis B control efforts and support alignment with the WHO 2030 elimination targets, a multifaceted, evidence-based approach is required. Routine adult serological screening should be incorporated into primary care services and high-risk occupational settings to identify individuals who lack protective hepatitis B surface antibodies. In parallel, booster vaccination strategies should be implemented for vulnerable populations, including young adults, healthcare workers, and ethnic minorities group with higher susceptibility, in accordance with international recommendations.

Catch-up vaccination initiatives should be further strengthened for adults born before the introduction of universal infant hepatitis B vaccination in 1989, with particular emphasis on ensuring equitable access across all socioeconomic groups. In addition, targeted health education and community outreach programmes are essential to address misconceptions regarding long-term immunity, especially among highly educated and single adults, as well as among minority populations where structural barriers may impede vaccine uptake. Finally, integrating hepatitis B surveillance into existing health information systems-through the routine monitoring of anti-HBs levels and long-term vaccine effectiveness-would provide critical data to guide adaptive and responsive public health policies. By prioritizing these measures, Malaysia can reduce immunity gaps, protect vulnerable populations, and move closer to the elimination of hepatitis B as a public health threat.

## Supplementary Information

Below is the link to the electronic supplementary material.

Supplementary Material 1

## Data Availability

The datasets used and/or analyzed are included in this published article. Also available from the corresponding author upon reasonable request.

## Abbreviations

NHMS: National Health and Morbidity Survey
HBV: Hepatitis B virus
WHO: World Health Organization
HCC: Hepatocellular carcinoma
EPI: Expanded Programme on Immunization
CMIA: Chemiluminescent Microparticle Immunoassay
CDC: Centers for Disease Control and Prevention
NHANES: National Health and Nutrition Examination Survey
CI: Confidence interval
aOR: Adjusted odd ratio
HLA: Human leukocyte antigen
