# Supplementary material for: Prevalence and factors associated with characteristics of hepatitis B susceptibility among vaccinated adults in Malaysia: a cross-sectional study
Source: Sci Rep. 2026 Mar 3;16:11919. doi: 10.1038/s41598-026-42115-9 (PMC13066411; doi:10.1038/s41598-026-42115-9)
Supplement: Supplementary file 1 — Supplementary Material 1 [file 41598_2026_42115_MOESM1_ESM.docx]

| **Variables**  **Supplementary Table S1:** Prevalence of susceptibility to HBV by sociodemographic variables among Malaysian adults and among vaccinated adults. | **Participant, n (%)** | **Estimated population, N** | **Susceptible to Hepatitis B** | | | **Susceptible to Hepatitis B among vaccinated adults** | | |
| --- | --- | --- | --- | --- | --- | --- | --- | --- |
|  |  |  | **Unweighted count, n** | **Estimated population, N** | **Prevalence (%)** | **Unweighted count, n** | **Estimated population, N** | **Prevalence (%)** |
| **Overall** | 4083(100.0) | 24,205,348 | 2649 | 15,155,978 | 62.9 (57.4, 68.0) | 548 | 3,403,770 | 22.9 (16.8, 30.5) |
| **Residential area** |  |  |  |  |  |  |  |  |
| Urban | 1891 (46.3) | 18,767,041 | 1478 | 11,840,323 | 63.4 (56.7, 69.5) | 346 | 2,868,305 | 15.0 (10.3, 21.5) |
| Rural | 2192 (53.7) | 5,438,307 | 1171 | 3,315,655 | 61.1 (52.9,68.6) | 202 | 535,464 | 9.8 (5.4, 16.9) |
| **Gender** |  |  |  |  |  |  |  |  |
| Male | 1923 (47.1) | 12,478,826 | 1198 | 7,650,783 | 61.6 (56.0, 67.0) | 249 | 1,692,885 | 13.4 (9.4, 18.8) |
| Female | 2160 (52.9) | 11,726,522 | 1451 | 7,505,195 | 64.2 (58.2,69.8) | 299 | 1,710,885 | 14.3 (10.2, 19.8) |
| **Age group** |  |  |  |  |  |  |  |  |
| 15-29 | 1257 (30.8) | 8,814,160 | 979 | 6,616,029 | 75.4 (67.6, 81.8) | 265 | 1,708,186 | 19.0 (13.5, 26.2) |
| 30-39 | 785 (19.2) | 5,304,485 | 533 | 3,326,749 | 62.8 (55.6, 69.5) | 122 | 761,199 | 14.2 (10.2, 19.4) |
| 40-49 | 685 (16.8) | 3,693,993 | 442 | 2,318,327 | 63.2 (55.3, 70.4) | 80 | 507,957 | 13.7 (7.9, 22.7) |
| 50-59 | 630 (15.4) | 2,991,937 | 348 | 1,418,467 | 47.7 (40.6, 55.0) | 48 | 242,391 | 7.9 (4.1, 14.7) |
| 60 years and above | 726 (17.8) | 3,400,772 | 347 | 1,476,408 | 43.4 (37.0, 50.1) | 33 | 185,035 | 5.4 (3.3, 8.6) |
| **Ethnicity** |  |  |  |  |  |  |  |  |
| Malay | 2494 (61.1) | 11,552,958 | 1807 | 8,770,773 | 76.0 (72.2, 79.5) | 379 | 2,398,174 | 20.5 (14.1, 28.8) |
| Chinese | 387 (9.5) | 5,449,457 | 111 | 1,709,323 | 31.4 (23.6, 40.0) | 24 | 343,797 | 6.1 ((3.3, 11.1) |
| Indian | 200 (4.9) | 1,526,953 | 155 | 1,142,202 | 74.8 (64.5, 82.9) | 19 | 170,258 | 11.3 (5.1, 22.9) |
| Other Bumiputera^a^ | 707 (17.3) | 2,716,832 | 429 | 1,725,768 | 64.2 (56.4, 71.4) | 117 | 414,812 | 15.1 (9.2, 23.9) |
| Others | 295 (7.2) | 2,959,148 | 147 | 1,807,913 | 62.0 (47.4, 74.7) | 9 | 76,728 | 2.6 (1.0, 6.5) |
| **Education level** |  |  |  |  |  |  |  |  |
| No formal education | 266 (6.5) | 1,352,333 | 123 | 764,811 | 57.6 (40.4, 73.1) | 7 | 10,533 | 5.8 (0.8, 32.7) |
| Primary education | 884 (21.7) | 4,776,596 | 488 | 2,488,707 | 52.5 (45.1, 59.8) | 71 | 359,521 | 7.5 (5.0, 11.1) |
| Secondary education | 1927 (47.2) | 10,608,786 | 1344 | 7,119,940 | 67.2 (61.4, 72.6) | 294 | 1,638,835 | 15.2 (10.9, 20.8) |
| Tertiary education | 1006 (24.6) | 7,161,323 | 694 | 4,580,799 | 64.0 (56.6, 70.9) | 176 | 1,384,367 | 18.9 (13.0, 26.7) |
| **Marital status** |  |  |  |  |  |  |  |  |
| Single | 1167 (28.6) | 7,675,557 | 877 | 5,481,842 | 71.8 (65.3, 77.6) | 245 | 1,563,971 | 20.1 (14.2, 27.5) |
| Married | 2564 (62.8) | 14,557,162 | 1584 | 8,802,936 | 60.7 (55.4, 65.7) | 281 | 1,716,772 | 11.6 (8.1, 16.4) |
| Widow(er)/Divorcee | 351 (8.6) | 1,969,974 | 187 | 868,545 | 44.1 (34.8, 53.8) | 22 | 123,027 | 6.1 (3.4, 10.9) |

**Note:**

**^a^ Other Bumiputera includes Bumiputera Sabah, Bumiputera Sarawak and Orang Asli**
